# Endophytic Fungi Produce Gibberellins and Indoleacetic Acid and Promotes Host-Plant Growth during Stress

**DOI:** 10.3390/molecules170910754

**Published:** 2012-09-07

**Authors:** Muhammad Waqas, Abdul Latif Khan, Muhammad Kamran, Muhammad Hamayun, Sang-Mo Kang, Yoon-Ha Kim, In-Jung Lee

**Affiliations:** 1 School of Applied Biosciences, College of Agriculture and Life Sciences, Kyungpook National University, Daegu 702-701, Korea; Email: agronomist89@gmail.com (M.W.); latifepm78@yahoo.co.uk (A.L.K.); m.kamran60@gmail.com (M.K.); kmoya@daum.net (S.-M.K.); kimyoonha@gmail.com (Y.-H.K.); 2 Department of Agriculture Extension, Government of Khyber Pakhtunkhwa, Bunir 19290, Pakistan; 3 Department of Botany, Kohat University of Science and Technology, Kohat 26000, Pakistan; 4 Department of Botany, Abdul Wali Khan University, Mardan 23300, Pakistan; Email: hamayun@awkum.edu.pk

**Keywords:** endophytic fungi, phytohormones, abiotic stress, mutualism

## Abstract

We isolated and examined two endophytic fungi for their potential to secrete phytohormones viz. gibberellins (GAs) and indoleacetic acid (IAA) and mitigate abiotic stresses like salinity and drought. The endophytic fungi *Phoma glomerata* LWL2 and *Penicillium* sp. LWL3 significantly promoted the shoot and allied growth attributes of GAs-deficient dwarf mutant *Waito-C* and Dongjin-beyo rice. Analysis of the pure cultures of these endophytic fungi showed biologically active GAs (GA_1_, GA_3_, GA_4_ and GA_7_) in various quantities. The cultures of *P. glomerata* and *Penicillium* sp. also contained IAA. The culture application and endophytic-association with host-cucumber plants significantly increased the plant biomass and related growth parameters under sodium chloride and polyethylene glycol induced salinity and drought stress as compared to control plants. The endophytic symbiosis resulted in significantly higher assimilation of essential nutrients like potassium, calcium and magnesium as compared to control plants during salinity stress. Endophytic-association reduced the sodium toxicity and promoted the host-benefit ratio in cucumber plants as compared to non-inoculated control plants. The symbiotic-association mitigated stress by compromising the activities of reduced glutathione, catalase, peroxidase and polyphenol oxidase. Under stress conditions, the endophyte-infection significantly modulated stress through down-regulated abscisic acid, altered jasmonic acid, and elevated salicylic acid contents as compared to control. In conclusion, the two endophytes significantly reprogrammed the growth of host plants during stress conditions.

## 1. Introduction

Salinity and drought are becoming more prominent and persistent throughout the World, posing great threats to sustainable agriculture production. Due to increasing global temperatures, the evaporation rate from soil is often altered, which cause saline conditions. Plants are sessile in nature and confronted with both biotic and abiotic stress conditions. Such stressful conditions produce reactive oxygen species (ROS) inside plants which cause cell death upon prolonged exposure. During stress conditions, ROS such as superoxide (O_2_^−1^), singlet oxygen (^1^O_2_), hydrogen peroxide (H_2_O_2_), and hydroxyl radical (OH) represent the free electrons leaked from electron transport chains in mitochondria and chloroplasts [1,2,3]. With the exception of H_2_O_2_, ROS cannot penetrate biological membranes. These are retained inside cells, signaling the cells to recruit local antioxidants to reduce ROS toxicity [4,5]. Both kinds of antioxidants, *i.e*., enzymatic and non-enzymatic, act to smoothly scavenge the ROS and prevent the adverse effects of free radicals [6,7]. Reduced glutathione, peroxidase, catalase and polyphenol oxidase are some of the key antioxidants which not only perceive oxidative stress but also counteract the stress by regulating their activities. To further cope with the negative effects of ROS, plants initiate physiological and biochemical changes depending on the varying ability of plant to perceive stimulus and transmit signals [7,8]. 

Plant hormones like abscisic acid (ABA), salicylic acid (SA) and jasmonic acid (JA) respond to abiotic stress stimuli and act as defense signaling substances [9]. In response to biotic and abiotic stresses, JA induces the biosynthesis of defense related-proteins and protective secondary metabolites [10,11]. Jasmonates can modulate many physiological events, such as resistance to pathogens and insects, pollen development, root growth and senescence [12]. Abscisic acid (ABA), on the other hand, is a ubiquitous phytohormone concerned with mediating stomatal closure [13] and regulating plant-growth and development during stress [9]. It is reported that drought resistance is acquired by inhibiting gibberellins (GAs) biosynthesis and increasing in ABA [14]. Salicylic acid (SA) is another phytohormone, playing its important roles in flower induction, growth and development, ethylene biosynthesis, stomatal behavior, and respiration in many plants [15]. SA plays an important role in biotic and abiotic stress whilst causing induced systemic resistance (ISR) against plant growth promoting fungi.

To mitigate stress while not compromising plant growth and yield has been suggested an ideal strategy. Plant growth-promoting fungal association has been perceived beneficial to host-plants even during stress conditions. Among fungi, endophytes grow within every plant organ without causing any disease symptoms [16]. Endophytes are symptomless microorganisms living inside host plant, that enhance host plant growth, improve nutrients uptake, reduce disease severity and enhance host tolerance to environmental stresses [17,18]. Besides being highly diverse in nature, these endophytes are a novel source of bioactive secondary metabolites [19,20,21]. Host-plants without endophyte-fungal association are devastated by the waves of extreme temperature, drought, salinity and pathogen attack [22]. Hence, productivity is frequently compromised in such situations. These endophytes fetch higher macro- and micro-nutrients like phosphorus, sulfur, calcium, magnesium and potassium. This capability has often been considered due to the potential of these endophytes to produce various biologically active metabolites and enzymes [23]. Among metabolites, plant hormones like GAs and auxin production is a new phenomenon in the endophytic fungi. Both GAs and auxin have been reported to play a pivotal role in plant growth, reproduction, metabolism and respond to various environmental cues. In last decade or so, it has been a known factor that these endophytic fungi, residing inside host confer abiotic stress tolerance [18,23,24]. However, the exact mechanism is still unexplored. In present study, we aimed to isolate, screen and identify such endophytic fungal strains which not only improve plant-growth but also extend greater stress tolerance to the host-plants. 

## 2. Results and Discussion

### 2.1. Endophyte Isolation and Initial Screening

We isolated 18 endophytes from roots of field grown cucumber plants. After a week, the isolated endophytes grown on PDA plates were grouped on the basis of morphological traits [25]. On the basis of morphological trait analysis, two endophytes were found different which were selected for further screening bioassay. To know the growth-promoting or inhibiting endophytic fungal strain, pure culture filtrates were applied to Dongjin-byeo (GAs producing normal cultivar) and *Waito-C* (GAs mutant and dwarf cultivar). Plant growth characteristics were recorded after one-week of treatment and the results are summarized in Table 1 and Table 2. In comparison to control (DDW applied plants) all the two strains were found growth-promoting.

**Table 1:** Effect of culture filtrate of isolated endophytic fungal strains on the growth of *Waito-C* rice.

Strain	SL (cm)	CC (SPAD)	SFW (g)	SDW (g)
**Control (GF)**	8.3 + 0.51 b	28.5 + 0.67 b	0.1386 + 0.1 b	0.0319 + 0.81 ab
**Control (DW)**	7.3 + 0.21 c	22.86 + 1.3 c	0.0986 + 0.07 c	0.0357 + 0.11 b
**CSH-5C**	9.3 + 0.35 a	29.43 + 0.89 b	0.155 + 0.104 a	0.0373 + 0.01 a
**CSC-1A**	9.6 + 1.6 a	31.4 + 0.36 a	0.1622 + 0.08 a	0.0405 + 0.109 a

SL = Shoot Length, GF = *Gibberella fujikuroi*, DW = Distilled Water, CC = Chlorophyll Content, SFW = Shoot Fresh Weight, SDW = Shoot Dry Weight. For each set of treatment, the different letter indicates significant differences at *p <* 0.05 levels as estimated by Duncan’s Multiple Range Test (DMRT).

The CSH-5C and CSC-1A significantly increased the shoot length, chlorophyll content, shoot fresh and dry weight of *Waito*-C as compared to control, *i.e*., *G. fujikuroi* culture filtrate applied plants, respectively. To confirm the preliminary screening experiment, we subjected these two bioactive strains (CSC-1A and CSH-5C) to know their effects on normal rice cultivar (Dongjin-byeo). Both strains again followed the same trend and increased the growth attributes, *i.e*., shoot length, shoot fresh weight, dry weight and chlorophyll content in comparison to controls (Table 2). In light of the significant results of the two strains, they were selected for identification, GAs analysis, host-plant interaction under salinity and drought stress mitigation.

**Table 2:** Effect of culture filtrate of isolated endophytic fungal strains on the growth of Dongjin-beyo rice.

Strain	SL (cm)	CC (SPAD)	SFW (g)	SDW (g)
**Control (DW)**	8.4 ± 0.44 c	15.26 ± 3.88 c	0.1094 ± 0.07 c	0.0357 ± 0.13 b
**CSH-5C**	11.5 ± 0.25 b	24.13 ± 1.95 a	0.1596 ± 0.104 a	0.0493 ± 0.06 a
**CSC-1A**	12.66 ± 1.2 a	19.96 ± 0.64 b	0.1283 ± 0.08 b	0.0395 ± 0.102 b

SL = Shoot Length, DW = Distilled Water, C.C = Chlorophyll Content, SFW = Shoot Fresh Weight, SDW = Shoot Dry Weight. Values with different letters in the same column in that group are significantly different at the 5% level by DMRT (Duncan’s Multiple Range Test). Values within the table refers to the mean ± SE (n = 5).

### 2.2. Identification and Phylogenetic Analysis

The fungal DNA of CSC-1A and CSH-5C was extracted for endophyte-identification. Internal transcribe spacer (ITS) regions of the endophytes was sequenced and phylogenetic analysis were carried out with the help of MEGA 5.0 software [26]. Sequences obtained were run through BLAST search program and related fungal sequences presenting the highest sequence similarity, query coverage and lowest E values were selected. BLAST search and phylogenetic tree results showed that CSC-1A and CSH-5C has 100% similarity with *Phoma glomerata* and *Penicillium* sp. respectively. The *Phoma glomerata* LWL2 (CSC-1A) and *Penicillium* sp. LWL3 (CSH-5C) sequence were submitted to NCBI Gene Bank for accession number (Supplementary information 1). The NCBI Gene Bank accession numbers of *Phoma glomerata* LWL2 and *Penicillium* sp. LWL3 were JX111911 and JX111910 respectively.

### 2.3. Analysis of Culture Filtrates for Screening Gibberellins

**Figure 1:**
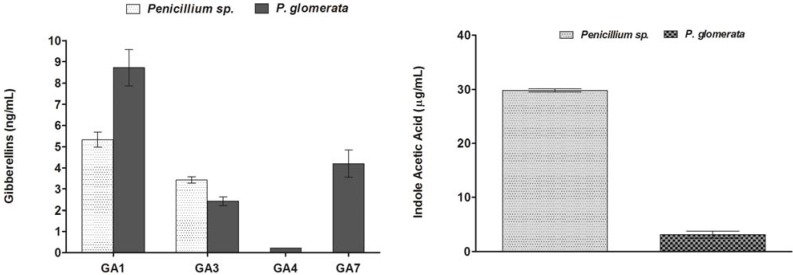
Gibberellins and indoleacetic acid production and quantification in the growing culture medium of *Penicillium* sp. and *P. glomerata.* The experiments were repeated three times.

To quantify the GAs production capability of *Penicillium* sp. (CSH-5C) and *P. glomerata* (CSC-1A), 250 mL of Czapek media was inoculated and kept on a shaking incubator for 7 days at 30 °C and 120 rpm. For GAs extraction and quantification the culture medium was centrifuged (2,500 × *g* at 4 °C) and 50 mL CF was further analyzed through GC/MS SIM. Four different GAs, both active and inactive, were found in the CF of *Phoma glomerata* (Figure 1; Supplementary Information 2 and 3).

Among physiologically active GAs, GA_1_ (8.720 ng mL^−1^), GA_3_ (2.420 ng mL^−1^) and GA_4_ (0.220 ng mL^−1^) were detected while the inactive included GA_7_ (4.200 ng mL^−1^). In case of *Penicillium* sp. only two active GA_1_ (5.33 ng mL^−1^) and GA_3_ (3.42 ng mL^−1^) were detected and no inactive GAs was found (Figure 1).

### 2.4. Quantification of IAA in Culture Filtrate

Both strains produced varying levels of IAA in their culture filtrate. The range of IAA production with or without tryptophan was found to be 3.89 ± 0.3 µg/mL in *P. glomerata*, while *Penicillium* sp. produced a significantly higher amount of IAA (29.8 ± 1.2 µg/mL). These endophytic fungal strains thus varied greatly in their inherent ability to produce IAA (Figure 1).

### 2.5. Response of Endophytes to Salinity and Drought Stress

**Figure 2:**
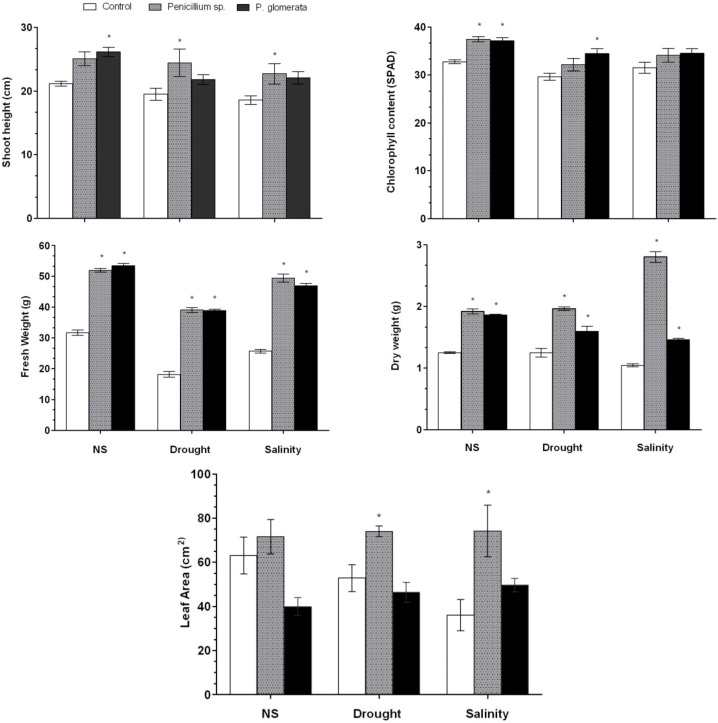
Growth attributes of the cucumber plants infested with the two endophytes, *i.e*., *Penicillium* sp. and *P. glomerata* with or without salinity and drought stress conditions. The growth parameters include, shoot height, shoot fresh weight, dry weight, chlorophyll content and leaf area. (*n* = 18). Each value is the mean ± SD of three replicates per treatment. NS stands for no-stress treatments. The ‘*’ indicates that values are significantly different from control (*p* < 0.05).

Because of the growth-promoting effects on the GAs mutant and normal rice cultivars, the symbiotic-association of endophytic fungi (*Penicillium* sp. and *P. glomerata*) with the cucumber plants under salinity and drought conditions was evaluated in growth chamber experiments. Cucumber plants were exposed to salinity (NaCl-140 mM) and drought stress (PEG-15% or −3.02 mPa of osmotic potential) for ten days. Cucumber plants treated with endophytes has higher shoot length than non-treated stressed plants. Under salinity stress, endophytes significantly increased the shoot length as compared to endophyte-free control. Application of endophyte also modulated the drought effects as we observed significantly higher shoot length as compared to control plants (Figure 2). Overall *Penicillium* sp. significantly ameliorated the plant height during salinity and drought stress while *P. glomerata* has increased the plant height significantly in inoculated plants under normal growth conditions followed by *Penicillium* sp. (Figure 2). Chlorophyll content was significantly higher in drought stressed plants inoculated with *P. glomerata*; however it was more pronounced in plants-inoculated with *Penicillium* sp. under normal growth conditions. 

**Figure 3:**
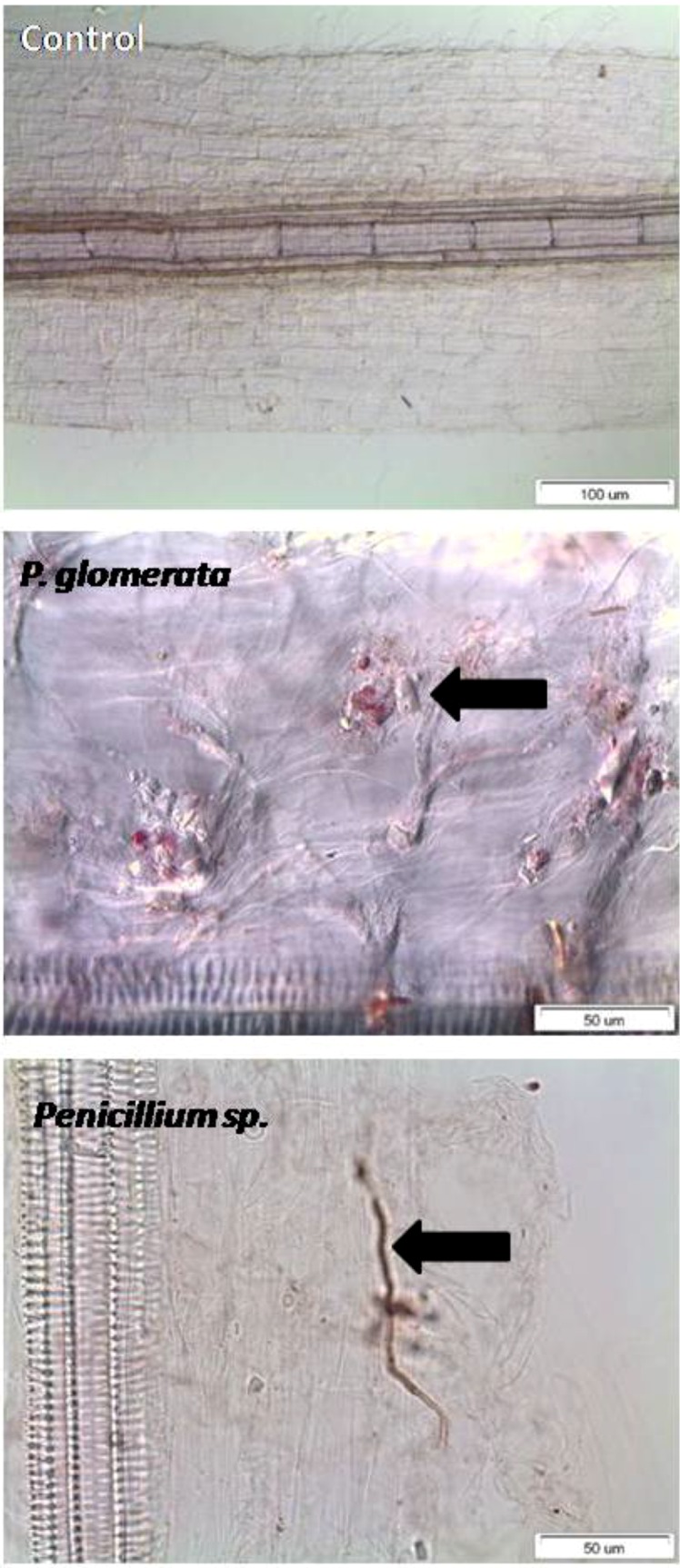
Endophyte associated with the roots of host cucumber plants. The control plant’s roots did not had any fungal colonization. Bar = 100 µm. *P. glomerata* occupied the pericycle regions of the roots in brownish yeast-like cells. Bar = 50 µm. The *Penicillium* sp. were found in the cortex region of the root. Bar = 50 µm.

Overall, the chlorophyll contents were significantly higher in endophyte-inoculated plants as compared to non-inoculated plants under stress conditions. Plant biomass, *i.e*., shoot fresh weight was significantly increased in cucumber plants associated with endophytes under both normal and stressful conditions. The same trend was repeated for the plant dry biomass under endophyte treatment and stress conditions (Figure 2). The leaf area of the cucumber plants was significantly higher in endophyte-infestation as compared to non-endophytic plants under stress conditions. The symbiotic association was also observed in plants inoculated with endophytes (Figure 3). It was found that both the endophyte species penetrated inside the root cortex of the cucumber plants. To the contrary, the roots of control plant did not have any colonization. 

### 2.6. Endophyte Benefit Ratio, and Dependency

**Figure 4:**
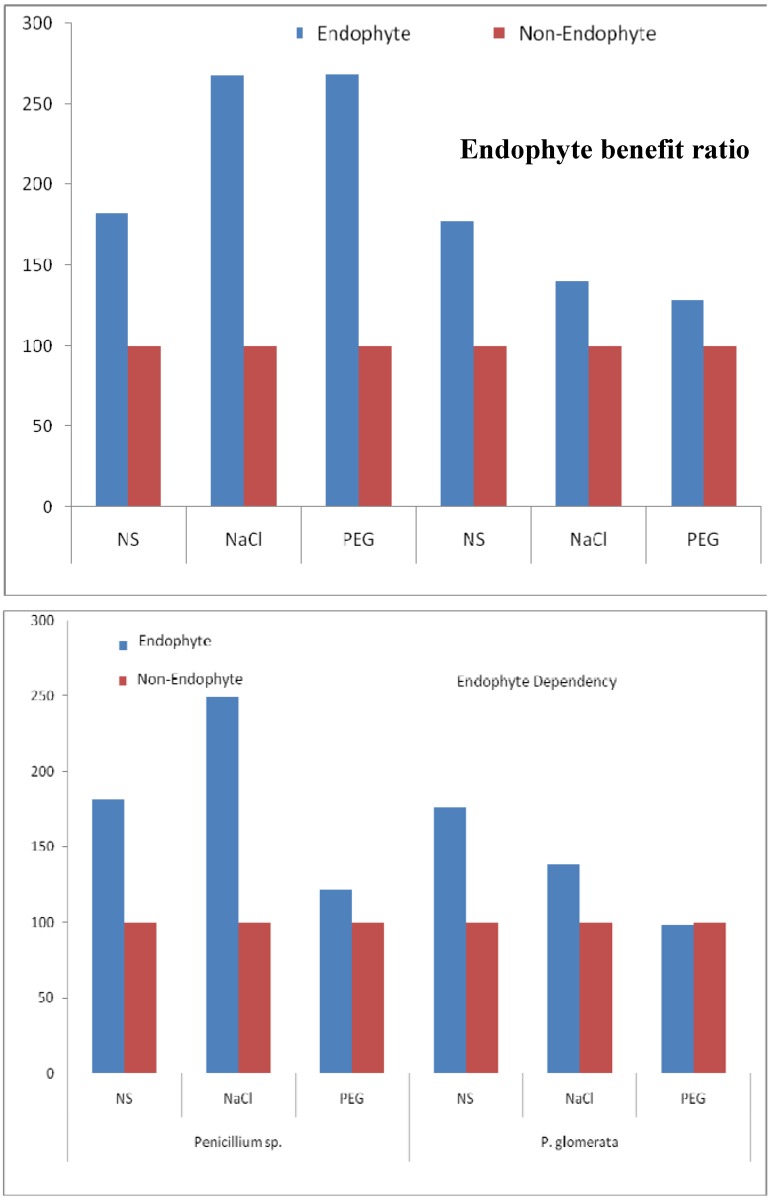
The endophyte-benefit ratio was calculated on the basis of shoot dry weight, the symbiosis of *Penicillium* sp. with cucumber was the most efficient as it increased the endophyte-benefit ratio under salt and drought stress. Each value is the three replicates per treatments.

The highest endophyte-benefit ratio with *Penicillium* sp., and *P. glomerata* under salt and drought stress was tested next. The symbiosis of *Penicillium* sp. with cucumber was the most efficient as it increased the endophyte-benefit ratio under salt and drought stress (Figure 4).

### 2.7. Symbiotic Association and Effect on Oxidative Stress during Abiotic stress

**Figure 5:**
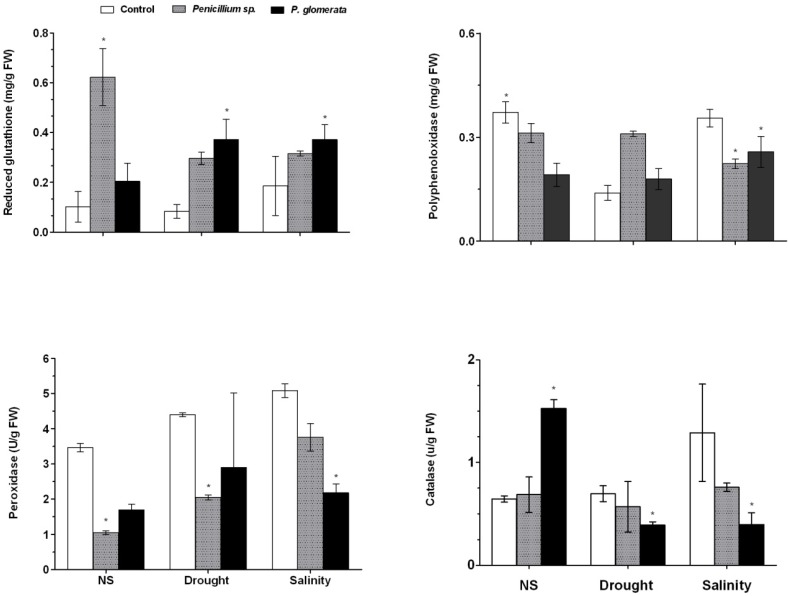
Effect of endophytic fungal association and abiotic stress treatment on the oxidative stress regulation. Antioxidants (reduced glutathione = GSH) and antioxidant enzymes (polyphenol oxidase = PPO; peroxidase = POD and catalase = CAT). Each value is the mean ± SD of three replicates per treatment. The ‘*’ indicates that values are significantly different from control (*p* < 0.05).

Under normal growth conditions, *Penicillium* sp. association increased the reduced glutathione (GSH) content. In drought, *P. glomerata* was more prominent in enhancing the GSH content. The polyphenol oxidase (PPO) activity was reduced. The results showed significant differences (*p* *<* 0.05) in response to endophyte-treatment under normal conditions (Figure 5). Application of *P. glomerata* and *Penicillium* sp. to cucumber plants during salinity stress accumulated low amount of PPO as compared to control plants (Figure 5). The results suggest that association of cucumber plants with endophytes had significant effects on the inhibition of catalase activities during salinity and drought stress. Moreover, under normal growth conditions, the association of *P. glomerata* has significantly elevated catalase activities than during the association of *Penicillium* sp. 

In cucumber plants, the peroxidase (POD) activity was significantly reduced during normal growth conditions and endophytic-fungal association. In case of drought and salt stress, endophytic fungal association reduced POD activity. *Penicillium* sp. significantly decreased the POD in drought stress and *P. glomerata* shows the same results in salt stress as compared to control (Figure 5).

### 2.8. Effect of Symbiotic Association on Host Plant Hormones ABA, JA and SA

**Figure 6:**
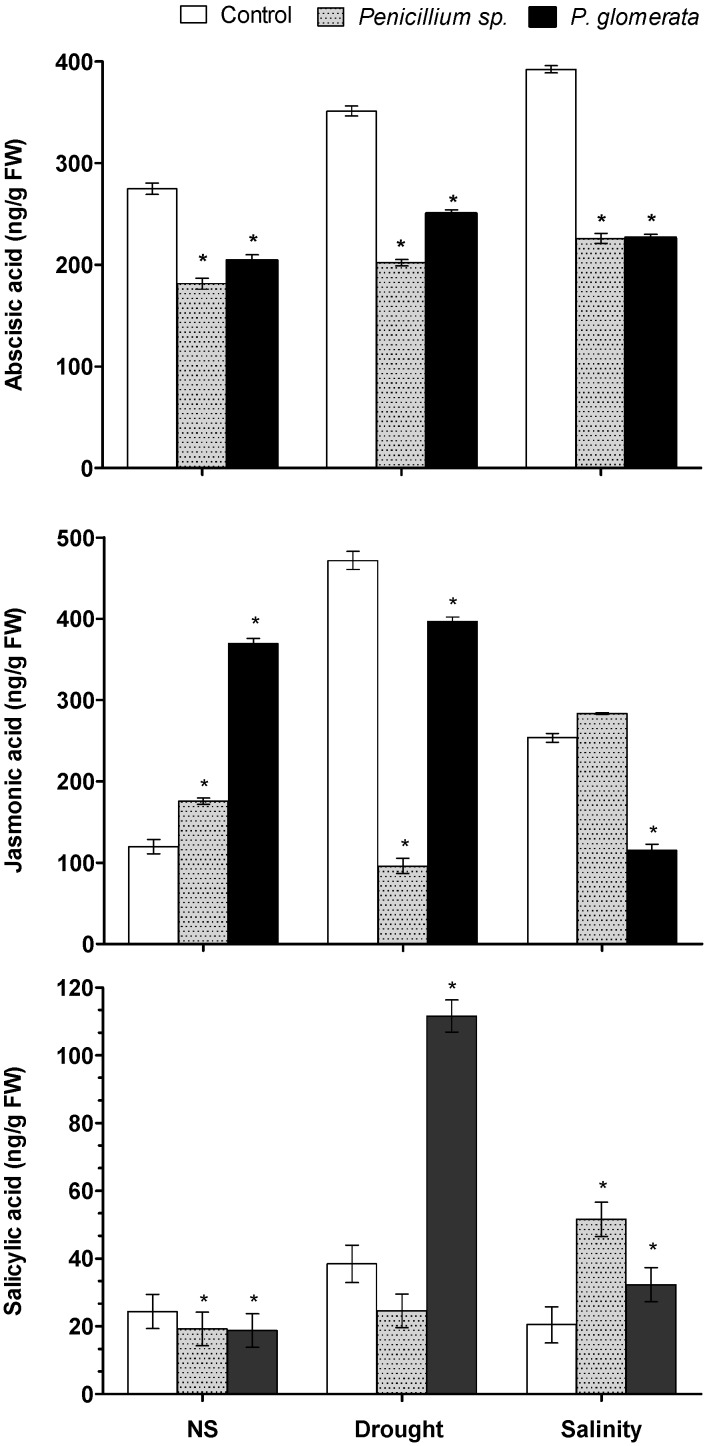
Phytohormonal (abscisic acid—ABA; salicylic acid—SA and jasmonic acid—JA) regulation of endophyte (*Penicillium* sp. and *P. glomerata*) and abiotic stress-treated plants. The quantities were calculated on the basis of peak area ratio with the standards using GC/MS SIM. (*n* = 3). Each value is the mean ± SD of three replicates per treatment. The ‘*’ indicates that values are significantly different from control (*p* < 0.05).

Application of endophytic fungi has highly significant results. The association reduced the stress-responsive endogenous ABA content. Among the two strains *Penicillium* sp. significantly reduced ABA content, followed by *P. glomerata*, under both salinity and drought conditions (Figure 6). In case of JA a different pattern was observed after endophyte application under normal and stress conditions. In case of control and drought stress, the plants with *P. glomerata* association had higher JA content as compared to non-infected control plants. Contrarily, under salinity stress, endophytes-inoculated plants had significantly low JA contents as compared to non-inoculated control plants (Figure 6).

Similarly *Penicillium* sp. produced a significantly lower amount of JA under drought stress and the highest under salinity stress. SA, on the other hand, was significantly lower in endophyte-inoculated plants as compared to control plants under normal growth conditions. During drought stress, the SA content was significantly higher in *P. glomerata* associated plants while in salinity stress, SA was high in *Penicillium* sp. as compared to non-infested control plants (Figure 6).

### 2.9. Elemental Analysis in Salinity Stress

**Figure 7:**
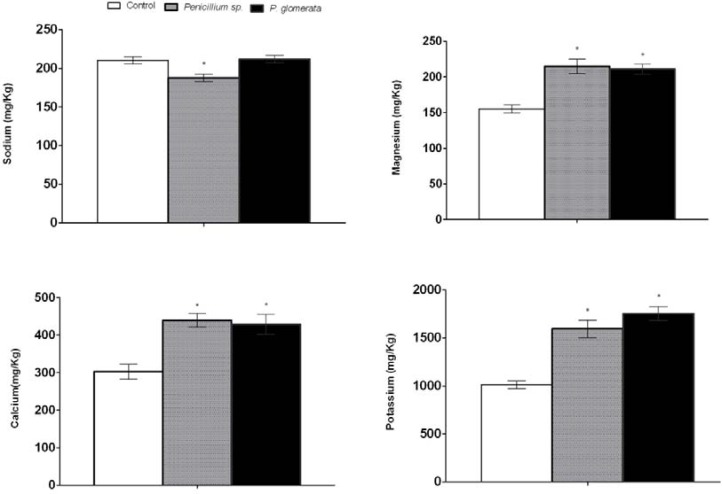
Sodium ion toxicity and essential micronutrient quantification (calcium, magnesium and potassium) of cucumber plants inoculated with or without endophytic fungi (*Penicillium* sp. and *P. glomerata*) under abiotic stress conditions. Each value is the mean ± SD of three replicates per treatment. The ‘*’ indicates that values are significantly different from control (*p* < 0.05).

We assessed the effect of endophytic-fungal association and salinity stress on the various macro and micronutrients. The results showed that the potassium (K) content was significantly higher in endophyte-inoculated plants than non-inoculated control plants. *P. glomerata* and *Penicillium* sp. had significantly higher K contents during salinity stress (Figure 7). Similarly, the calcium (Ca) level was significantly higher in *Penicillium* sp. and *P. glomerata* associated plants than non-associated control plants under salinity stress. It suggests that Ca signaling was significantly higher in endophyte treated plants. A same trend of higher magnesium content was also observed in endophyte-associated plants than control. However, it is worth mention that endophyte-infested plants have significantly low levels of sodium toxicity as compared to non-endophytic fungal plants. 

Similarly our results revealed that control plants under salinity stress showed lower K^+^/Na^+^, Mg^2+^/Na^+^ and Ca^2+^/Na^+^ ratios than those of endophyte-inoculated ones. Although these ratios were different among the two strains, however they were significantly higher than in non-inoculated plants and both strains proved their stress modulation under salt stress (Figure 7).

Symbiotic-association always exists between plants and endophytic fungi in natural ecosystems [27]. A plant endophytic fungus produces many natural bioactive compounds with great importance in agriculture, medicine and the food industry [28]. Endophytic fungi offer an important role in protection of plants and making plants more fit to cope with biotic and abiotic stress tolerance, decreasing water consumption and increasing biomass [16,29]. Endophytic fungi are promising sources of organic substances which can enhance plant growth; therefore in this experiment we isolated two strains from field grown cucumber plants and investigated their effect on plant growth-promotion.

The CF of fungal isolates was initially applied to *Waito-*C rice and Dongjin-beyo for the screening experiments. The results of mutant *Waito*-C rice (with blocked C13 hydroxylation pathway) and normal GA biosynthesis Dongjin-byeo are in conformity with the previous findings [30]. Thus, it helped us in accurate identification of endophytic fungi having potential to produce plant growth-promoting hormones. Mutant rice was treated with uniconazole to further suppress GAs biosynthesis which helped in detection of a very small amount of GAs present in culture [31]. In the present study, we observed that the CF of *Penicillium* sp. and *P. glomerata* significantly promoted the shoot growth and allied attributes of mutant *Waito-*C and normal Dongjin-beyo. The CF of the two selected endophytes was analyzed for GA production capability using GC MS/SIM [32,33]. The repetition of our experiment and correlation of GAs detection with the corresponding deuterated GAs standards further helped to confirm our findings of GAs production. Several researchers [34,35] have reported the plant growth-promoting characteristics of endophytic fungi mostly associated with roots and capable of secreting secondary metabolites, including phytohormones [25]. 

Endophytic fungi help the host plants respond to the varying environments by regulating plant growth and development using bioactive substances and sharing mutualistic genes which can work together with the hosts’ for better performance [16,36]. Salt and drought stress inhibits growth, start senescence and causes death in response to prolonged exposure [37,38]. Both salt and drought stress causes reduction in plant biomass, water potential and increase cellular membrane injury [39]. In the present study, the association of *Penicillium* sp. and *P. glomerata* resulted in higher shoot growth, plant biomass and chlorophyll content. The endophyte was secreting bioactive GAs like GA_3_ in its growing medium whilst the said hormone, if applied exogenously, displays ameliorative effects on plant growth with or without stress conditions [39]. A similar behaviour of an endophyte producing auxin was also reported [25] in which indoleacetic acid-producing endophytic fungi enhanced rice plant growth under salinity, drought and temperature stress. Thus, previous findings strongly support our results [25,40,41].

The present study indicated that application of NaCl induced significant increases in Na+ and decreases in K^+^, Mg^2+^ and Ca^2+^ levels in the root system of non-endophyte plants. High salt (NaCl) uptake competes with the uptake of other nutrient ions, especially K^+^, causing deficiency in K^+^ and other ions. The ion deficiencies develop a nutritional imbalance [42]. The current study showed that endophyte-inoculated plants contained significantly higher levels of K^+^, Mg^2+^ and Ca^2+^ ions, particularly in case of *Penicillium* sp. and *P. glomerata*, than non-inoculated plants under salt stress conditions. Previously, fungi were reported for the same function by enhancing nutrient uptake in infected plants under salinity conditions [43]. Our findings and previously reports suggest greater salt tolerance in the presence of endophytes which may be due the improvement in plant nutrition balance under stress. The endophyte might inhibit the uptake of Na^+^ or prevent its transport to other plant parts whilst producing bioactive substances.

During stress, the plants have developed an impressive array of non-enzymatic and enzymatic antioxidant systems, whose function is to counteract the ROS toxicity in their cells. Indeed, low ROS concentration is required for signaling, growth and development, while high concentrations are detrimental to cells [44]. These enzymes are involved in the removal of ROS either directly (catalases, and peroxidases) or indirectly through the regeneration of the two major redox molecules in the cell, ascorbate and glutathione. Higher the accumulation of these antioxidants reveals higher amount of stress in the plants [45]. CAT, POD and PPO activities were significantly lowered in endophyte and stress-treated plants as compared to control plants. These enzymes help the plants to eliminate H_2_O_2_ from mitochondria and microbodies and can regulate responses to stress. Previously, it has been suggested that increase in enzymatic activities causes the reduction of plant growth [6]. However, we observed altered levels, which indicate lesser amount of stress experienced by plants with endophytes.

Under stress conditions, plant hormone signaling is an important strategy during abiotic stress. Phytohormones like jasmonic and salicylic acid are the key regulators in plant defense. These can act synergistically or antagonistically with each other. Since small subsets of genes are affected by both ethylene and JA signals, therefore the interaction between these two pathways was likely to be downstream. Elevated levels of JA have been reported in various crop plants after excursion of abiotic stresses like salinity, drought [46] and herbivory [47]. In present study, we found that endophyte-inoculation and abiotic stress can alter to JA response. SA, on the other hand, was significantly higher in endophyte-infested plants than control. This altered level of JA, elevated SA and reduced synthesis of ABA might be correlated with the low level of stress convened to the endophyte-treated plants than control plants. 

## 3. Experimental

### 3.1. Sample Collection and Endophyte Isolation

Mature and healthy cucumber plants were selected from samples grown under field conditions in the vicinity of Kyungpook National University, Daegu South Korea. Plants were immediately stored in plastic bags and brought to the laboratory for further processing. Prior to isolation cucumber plant roots were sterilized according to [24,25] and grown on Hagem media. Imprints of roots were also taken on Hagem media plates to ensure sterilization. The fungal spots appearing on Hagem media plates were transferred into new potato dextrose agar (PDA). 

### 3.2. Endophyte Identification

The endophytes were inoculated in Czapek broth media [48] for seven days (shaking at rpm 120, temperature 28 °C). After incubation, the broth was vacuum filtered using sterilized whatman filter paper no 2. Fresh mycelium was collected and fungal genomic DNA was extracted using the SolGent Fungus Genomic DNA Extraction Kit (Cat No. SGD64-S120; SolGent Co., Daejeon, Korea) following the manufacturer’s protocol. We obtained endophytic fungal sequences by using ITS1 (5′-TCC GTA GGT GAA CCT GCG G-3′) and ITS4 (5′-TCC TCC GCT TAT TGA TAT GC-3′)] [27]. The BLAST search program [49] was used to compare the nucleotide sequence similarity of ITS region of related fungi. The closely related sequences obtained were aligned through CLUSTAL W using MEGA version 4.0 (Tempe, AZ, USA) and a maximum parsimony trees were constructed for the two strains using the same software. The bootstrap replications (1K) were used as a statistical support for the nodes in the phylogenetic tree.

### 3.3. Gibberellins Deficient Mutant and Normal Rice Bioassay

For initial screening bioassay mutant gibberellins biosynthesis pathway *Waito-C* and normal gibberellins biosynthesis Dongjin-byeo cultivars were used. The experiment comprises of application of pure culture filtrate of different strains with positive control (culture of wild type *G. fujikuroi* KCCM 26329) and negative control (double distilled water—DDW). The culture filtrate (CF) obtained after mycelium extraction was concentrated in freeze dryer (Virtis Freeze Dryer; Gardiner, NY, USA) for 4 days and used for screening bioassay. Prior to bioassay the seeds of *Waito-c* and Dongjin-byeo were surface sterilized with 5% sodium hypochlorite. To obtained uniform seedling the sterilized seeds were transferred into petri dishes moistened with 5 mL autoclaved DDW and keep for germination for 4 days at 28 °C. The uniform seedlings were transferred into small pots with autoclaved 0.8% agar water media into growth chamber (day/night cycle: 14 h—28 °C ± 0.3;10 h—25 °C ± 0.3; relative humidity 70%; 18 plants per treatment) for 14 days. Concentrated pure culture filtrate were diluted with 1 mL autoclaved DDW and 100 µL was applied after seven-days on the apex of each seedling and plant growth attributes were measured after one week of CF application.

### 3.4. Gibberellins Analysis of Pure Culture

The CF of selected growth-promoting strains (two) was subjected to gibberellins analysis following the modified protocol of [50]. The culture medium (50 mL) was used to extract and purify GAs and the pH of the CF was adjusted to 2.5 using 6 N HCl. It was partitioned with ethyl acetate (EtOAc). Before partitioning, deuterated GAs internal standards (20 ng; [17,17–^2^H_2_] GA_1_, GA_3_, GA_4_, GA_8_, GA_12_ and GA_24_) were added in the CF. Tritiated Gas, *i.e.*, [1,2–^3^H_2_] GA_9_ and [1,2–^3^H_2_] GA_20_ were also added (obtained from Prof. Lewis N. Mander, Australian National University, Canberra, Australia). The organic layer was vacuum dried and added with 60% methanol (MeOH) while the pH was adjusted to 8.0 ± 0.3 using 2 N NH_4_OH. The CF was subjected to chromatographic and mass spectroscopy techniques for identification and quantification of GAs (Supplementary Information 2 and 3).

### 3.5. IAA Analysis of Pure Culture

Selected strains were inoculated in Czapek media with tryptophan (0.1 g/L) or without tryptophan incubated at 30 °C for 7 days to allow the fungi for the production of IAA. The media were vacuum filtrate and the pure culture filtrates were analyzed and quantified with a high performance liquid chromatography (HPLC) system [51,52].

### 3.6. Host Plant and Endophytes Association in Salinity and Drought Stress

Selected fungal strains were tested in abiotic stress condition, *i.e.*, salinity (140 mM) and drought stress (15% PEG). The experiment was carried out in completely randomized block design and treatment comprised of control with and without salinity and drought stress, endophytic fungi (two strains) with and without salinity and drought stress. Cucumber seeds were surface sterilized and incubated for 4 days at 28 °C to get uniform seedlings. Two seedlings were transferred to each sterile pot filled with autoclaved horticulture soil having composed of peat moss (13–18%), perlite (7–11%), coco-peat (63–68%) and zeolite (6–8%), with macro-nutrients present as: NH_4_—90 mg Kg^−1^; NO_3_—205 mg Kg^−1^; P_2_O_5_—350 mg Kg^−1^ and K_2_O—100 mg Kg^−1^ with growth chamber condition (day/night cycle: 14 h—28 °C ± 0.3; 10 h—25 °C ± 0.3; relative humidity 70%). Seedlings were inoculated with 200 mL of culture filtrate having mycelia of each fungal strain after 10 days of establishment in pots. The plants were lifted for 20 days in normal condition to properly make mutualistic relationship and then drought and salinity stress were induced. The abiotic stress was maintained for 8 days and every time 200 mL of 140 mM NaCl and 15% PEG was applied during the course of stress. After a week of stress condition the plant growth characteristics were measured and plant samples were immediately stored in −70 °C. 

Microscopic analysis of the host-endophyte symbiosis was performed on a light microscope (Olympus BX50, Tokyo, Japan). Cucumber roots inoculated with endophyte were sectioned and treated with sodium hypochlorite (2.5%) for 10 min for clarification. Experimental conditions were kept aseptic during analysis. Inoculated roots were treated with 20% KOH for 24 h and rinsed with autoclaved DDW. The roots were then acidified with 10% HCl, stained overnight using 0.8% tryptophan blue and 95% lactic acid. Finally, the roots were destained in 95% lactic acid for 24 h. The roots pieces were then subjected to light microscope (Stemi SV 11 Apo, Carl Zeiss, Jena, Germany).

### 3.7. Hormonal Analysis (ABA and JA) of Host Plant

The extraction of endogenous ABA was carried out according to the method of [53]. The extracts were dried and methylated by adding diazomethane. Analyses were done using a GC-MS SIM (6890N network GC system, and 5973 network mass selective detector (Agilent Technologies, Palo Alto, CA, USA). For quantification, the Lab-Base (ThermoQuset, Manchester, UK) data system software was used to monitor responses to ions of *m/z* 162 and 190 for Me-ABA and 166 and 194 for Me-[^2^H_6_]-ABA.

The endogenous JA level was extracted according to the protocol of [54]. The extracts were analyzed by GC-MS SIM. The amount of endogenous JA was calculated from the peak areas of endogenous JA in comparison with the corresponding standards. Two replicates per treatments were used for determination of JA and ABA.

The SA was extracted and quantified according to the protocol developed by [55,56]. High Performance Liquid Chromatography (HPLC) analyses were carried out on a Shimadzu instrument equipped with a fluorescence detector (Shimadzu RF-10AXL, excitation and emission, 305 and 365 nm, respectively) and fitted with a C18 reverse phase HPLC column (HP Hypersil ODS, particle size 5 µm, pore size 120 Å, Waters). The flow rate was maintained 1.0 mL/min.

### 3.8. Antioxidants Activities of Host Plant

Reduced glutathione (GSH) content was measured according to the method of [57]. Absorbance was determined at 412 nm (T60 UV VIS Spectrophotometer, Leicester, UK), and the GSH concentration was calculated by comparing with a standard curve. The experiment was repeated thrice. 

For catalase activity and protein determination, the leaves were homogenized in 50 mM Tris–HCl buffer (pH 7.0) containing 3 mM MgCl_2_, 1 mM EDTA, and 1.0% PVP and then centrifuged at 2,500 × g for 15 min at 4 °C; the supernatant was used for biochemical analysis. All parameters were expressed as activity per milligram protein. Catalase activity was assayed by the method of [58]. Catalase activity was estimated by the decrease in absorbance of H_2_O_2_ at 240 nm, and 1 U of catalase was defined as micrograms of H_2_O_2_ released per milligram protein per minute.

Peroxidase and polyphenoloxidase activity were measured as described by [59] with some modification. The amount of purpurogallin formed was determined by the absorbance at 420 nm. The same assay mixture as that of peroxidase without H_2_O_2_ was used to assay the activity of polyphenoloxidase. One unit of peroxidase and polyphenol oxidase was defined as an increase of 0.1 U of absorbance.

### 3.9. Elemental Analysis

The fresh plants materials were lyophilized and ground into fine powder form using a grinder. Elements, Na^+^, K^+^, Ca^2+^ and chloride ion were analyzed in the shoot dry tissues using inductively coupled plasma optical emission spectroscopy (ICP-OES, Varian Vista-PRO RL, Palo Alto, CA, USA) after digestion with concentrated HNO_3_.

### 3.10. Endophyte Benefit (%), Endophyte Dependency

For shoot dry weight the Endophyte benefit (%) was calculated using the following equations [60]: 


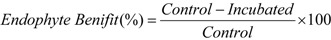


Endophytes dependency (ED) of the cucumber plants was calculated according to [61] as:


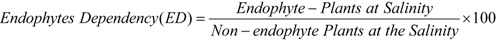


### 3.11. Statistical Analysis

The experiment was performed in RCBD and each treatment replicated six times. Data were analyzed in DMRT using SAS (version 9.2, Cary, NC, USA) and ANOVA and Bonferroni test was performed to separate the means where applicable using Graph Pad Prism 5.0 (San Diego, CA, USA).

## 4. Conclusions

Endophyte-infected plants showed low signs of the adverse effects of drought and salinity whilst the symbiotic-association enhanced the growth parameters of cucumber plants. The findings of the current study reveal that such endophytic fungal interactions can improve the quality and productivity of economically important crop species. However, the favorable role of these fungal strains still needs to be investigated under field conditions while assessment of transcriptomic regulation would help us to understand the mechanisms involved in stress tolerance.

## Supplementary Materials

Supplementary materials can be accessed at: http://www.mdpi.com/1420-3049/17/9/10754/s1.
