# Supplementary material for: Endophytic Fungi Produce Gibberellins and Indoleacetic Acid and Promotes Host-Plant Growth during Stress
Source: Molecules. 2012 Sep 7;17(9):10754–73. doi: 10.3390/molecules170910754 (PMC6268353; doi:10.3390/molecules170910754)
Supplement: Supplementary file 1 [file molecules-17-10754-s001.pdf]

Supplementary Information 1

Identification of endophytic fungal isolates. Endophytic fungi were indentified through the phylogenetic analysis of 18S and 28S rDNA sequences using maximum parsimony method with related fungi. The sequence analysis showed that the endophytic fungal isolates are new strains of *Penicillium* sp., and *Phoma glomerata.*

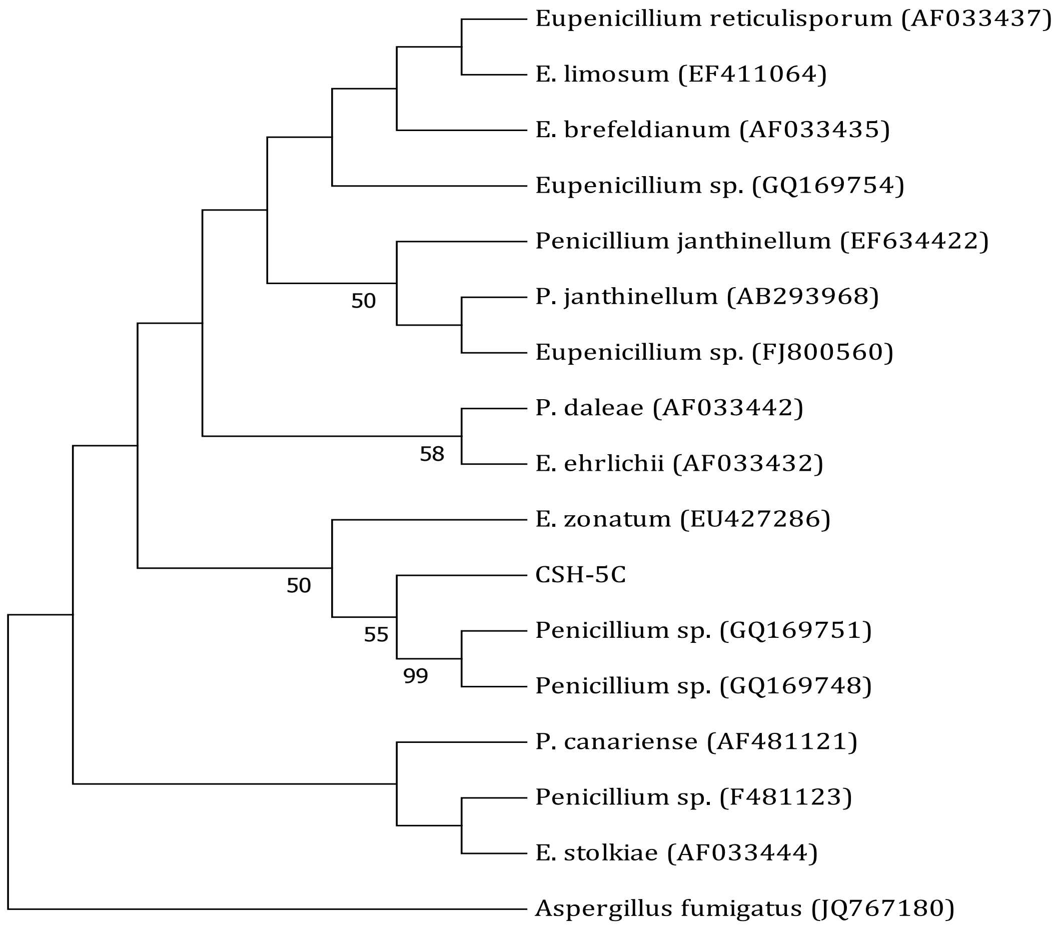

A.

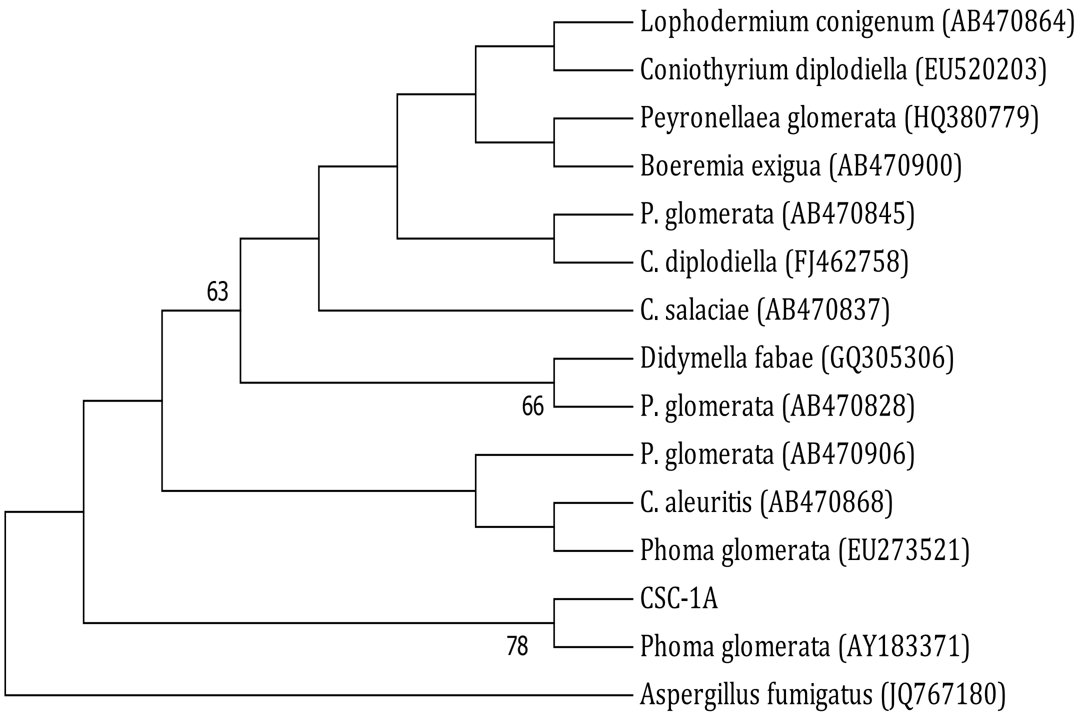
Lophodermium conigenum (AB470864)
Coniothyrium diplodiella (EU520203)
Peyronellaea glomerata (HQ380779)
Boeremia exigua (AB470900)
P. glomerata (AB470845)
C. diplodiella (FJ462758)
C. salaciae (AB470837)
Didymella fabae (GQ305306)
P. glomerata (AB470828)
P. glomerata (AB470906)
C. aleuritis (AB470868)
Phoma glomerata (EU273521)
CSC-1A
Phoma glomerata (AY183371)
Aspergillus fumigatus (JQ767180)
63
66
78

B.

Supplementary Information 2

GC/MS-SIM analysis of HPLC fractions of pure culture filtrate of endophyte.

HPLC fraction No /RT	RT [a]	Sample/Standard	GAs [b]	KRI [c]	*m/z* (%, relative intensity of base peak) [d]		
11~15	24.3	sample	GA_1	2674	506(100)	491(13)	313(17)
		standard	$[^2H_2]$ GA_1	2674	508(100)	493(15)	315(19)
11~15	25.48	sample	GA_3	2692	504(100)	489(8)	370(9)
		standard	$[^2H_2]$ GA_3	2692	506(100)	491(10)	372(11)
34~35	24.31	sample	GA_4	2506	284(100)	225(80)	289(70)
		standard	$[^2H_2]$ GA_4	2506	286(100)	227(76)	291(71)
37~38	23.49	sample	GA_9	2305	298(100)	270(78)	227(48)
		standard	$[^2H_2]$ GA_9	2305	300(100)	272(77)	229(48)

[a] RT: Retention time (in min); [b] GAs: Gibberellins; [c] KRI: Kovats retention indices; [d] Identified as methyl ester trimethylsilyl ether derivatives by comparison with reference spectra and KRI data as elucidated by Gaskin and MacMillan (1991). Gibberellins are identified with three ions and quantified by first ion with comparison of labelled standards. About 50 µL of CF extract of CSH-6H results in various HPLC fractions.

Supplementary Information 3

GC/MS Peaks of the CF of the Endophyte and standard

GA_1 Spectra

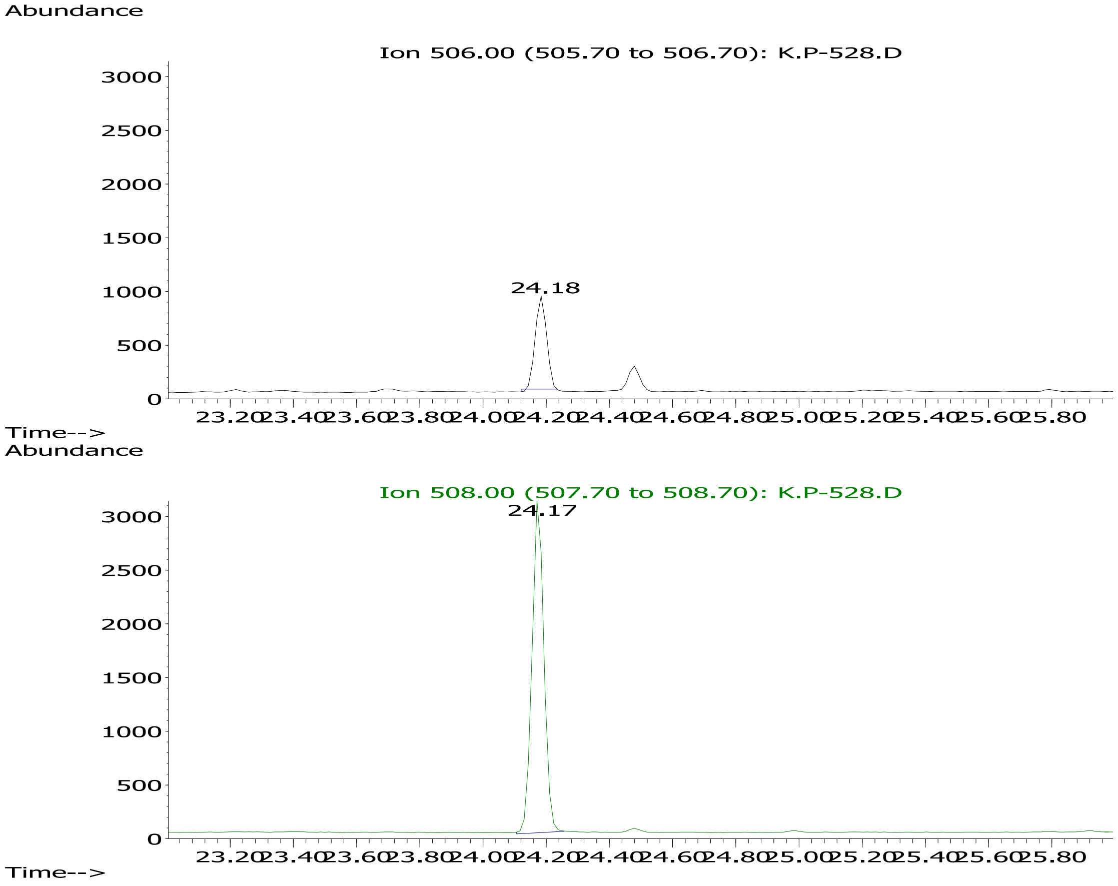

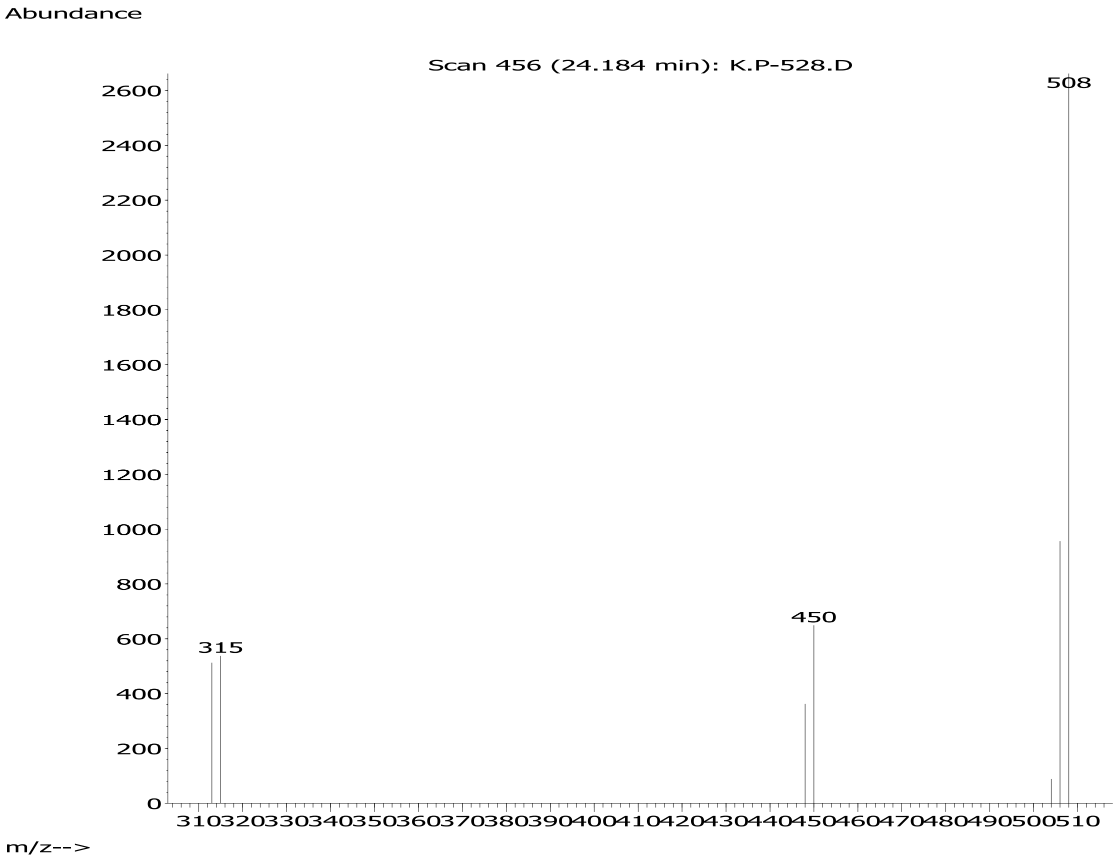
Abundance
Scan 456 (24.184 min): K.P-528.D
2600
2400
2200
2000
1800
1600
1400
1200
1000
800
600
400
200
0
315
450
508
310320330340350360370380390400410420430440450460470480490500510
m/z-->

GA3 Spectra

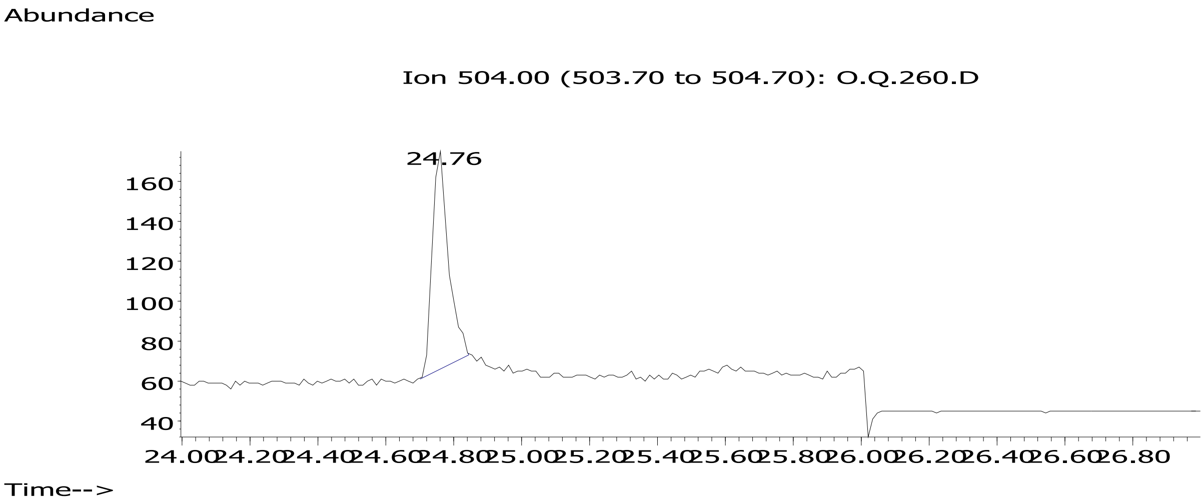
Abundance
Ion 504.00 (503.70 to 504.70): O.Q.260.D
24.76
160
140
120
100
80
60
40
24.00 24.20 24.40 24.60 24.80 25.00 25.20 25.40 25.60 25.80 26.00 26.20 26.40 26.60 26.80
Time-->

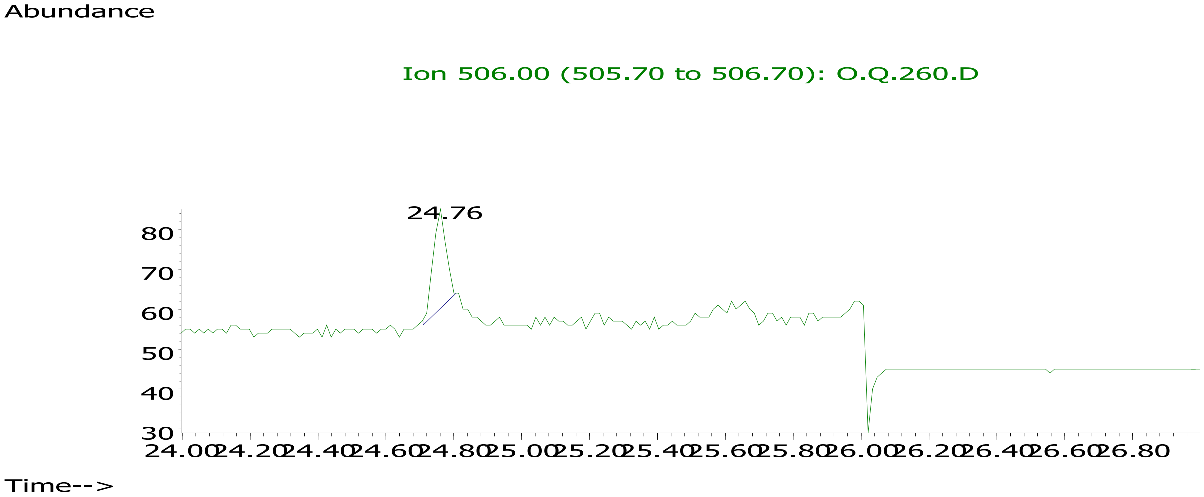
Abundance
Ion 506.00 (505.70 to 506.70): O.Q.260.D
24.76
80
70
60
50
40
30
24.00 24.20 24.40 24.60 24.80 25.00 25.20 25.40 25.60 25.80 26.00 26.20 26.40 26.60 26.80
Time-->

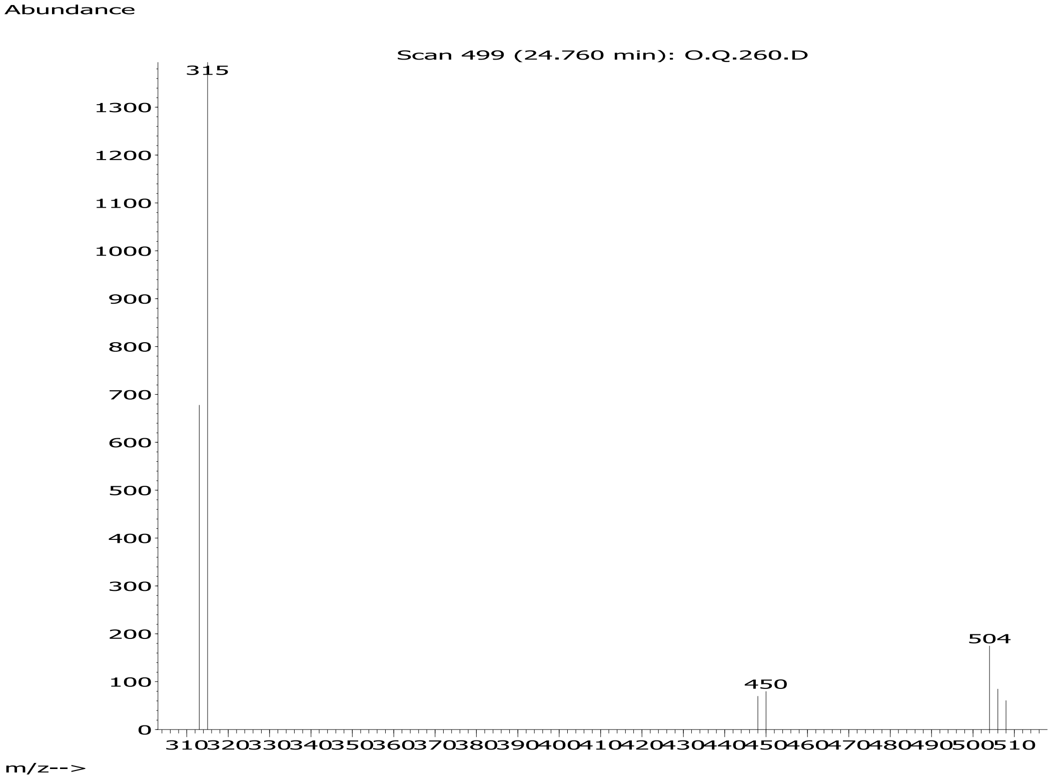
Abundance
Scan 499 (24.760 min): O.Q.260.D
315
450
504
1300
1200
1100
1000
900
800
700
600
500
400
300
200
100
0
310 320 330 340 350 360 370 380 390 400 410 420 430 440 450 460 470 480 490 500 510
m/z-->

GA$_9$ Spectra

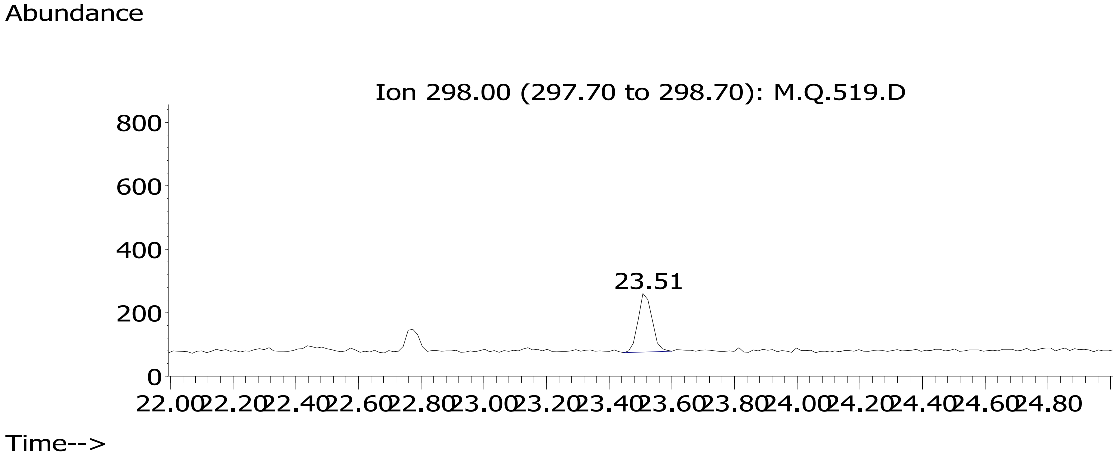
Abundance
Ion 298.00 (297.70 to 298.70): M.Q.519.D
23.51
Time-->

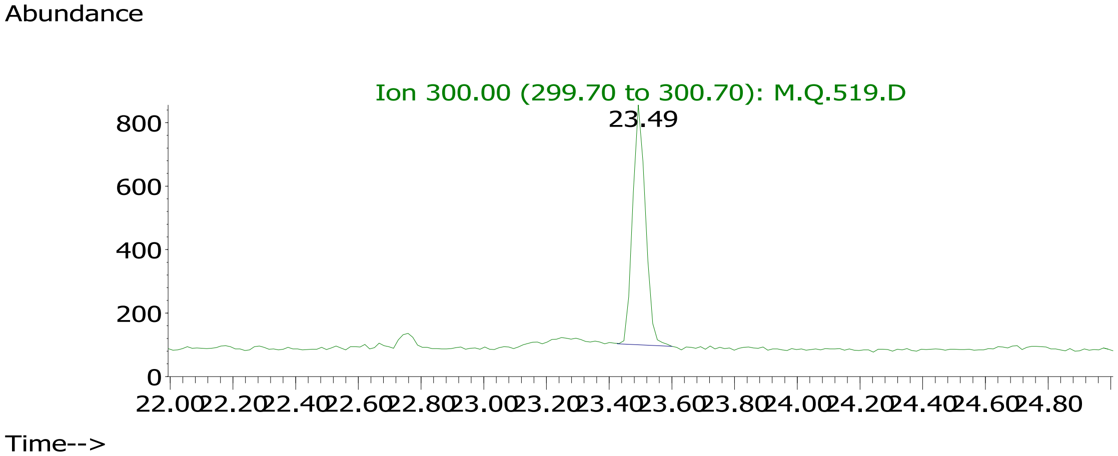
Abundance
Ion 300.00 (299.70 to 300.70): M.Q.519.D
23.49
Time-->

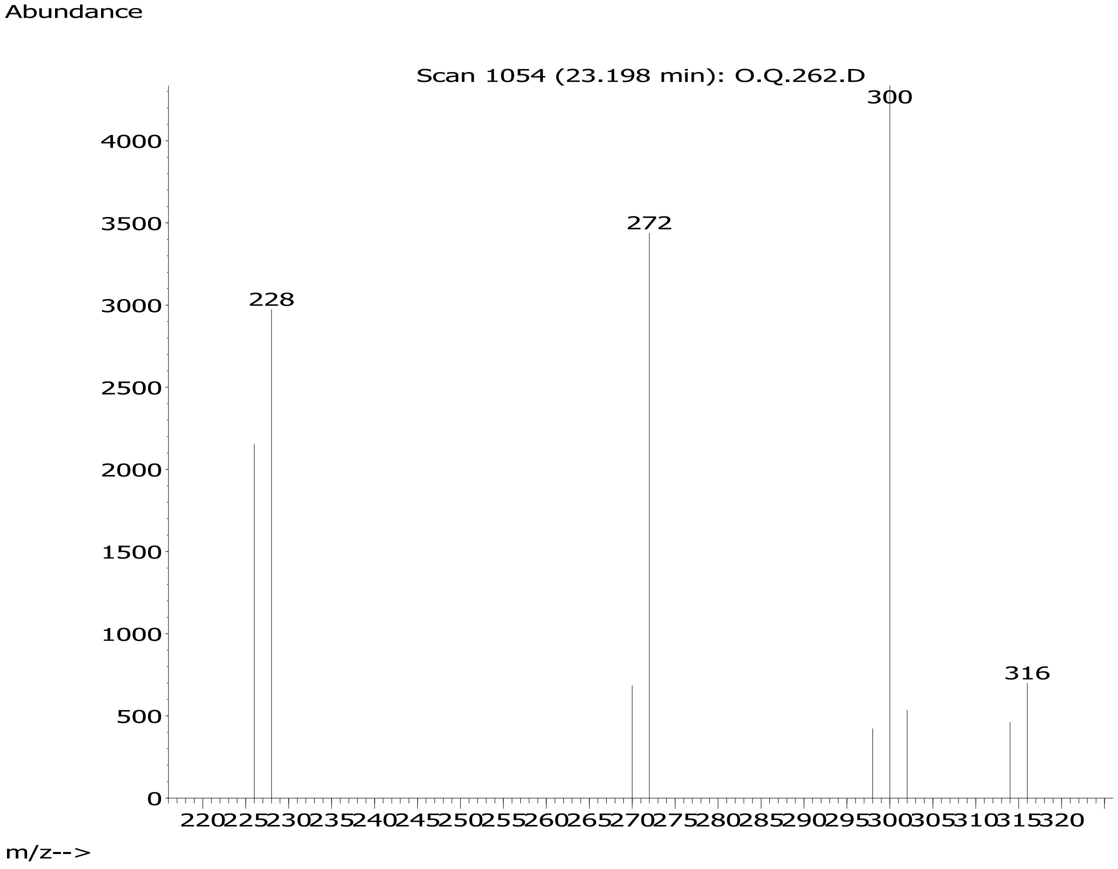
Abundance
Scan 1054 (23.198 min): O.Q.262.D
300
272
228
316
m/z-->

Supplementary Information 4

GC/MS–SIM conditions used for analysis and quantification of the Gibberellins.

Equipment	Hewlett-Packard 6890, 5973N Mass Selective Detector
Column	HP-1 capillary column (30 m × 0.25 mm i.d. 0.25㎛ film thickness) (J & W Scientific Co., Folsom, CA, USA)
Carrier gas	He (40 ㎖/min.); head pressure of 30 kPa
Source temperature	250 °C
Oven conditions	GA: 60 °C (1 min.) → 15 °C /min. → 200 °C (1 min.) → 5 °C /min. → 285 °C (5 min.)
Injector temperature	200 °C
Ionizing voltage	70 ev
